# Supplementary figures and images for: GATA2 participates in protection against hypoxia-induced pulmonary vascular remodeling
Source: PLoS One. 2024 Dec 31;19(12):e0315446. doi: 10.1371/journal.pone.0315446 (PMC11687799; doi:10.1371/journal.pone.0315446)

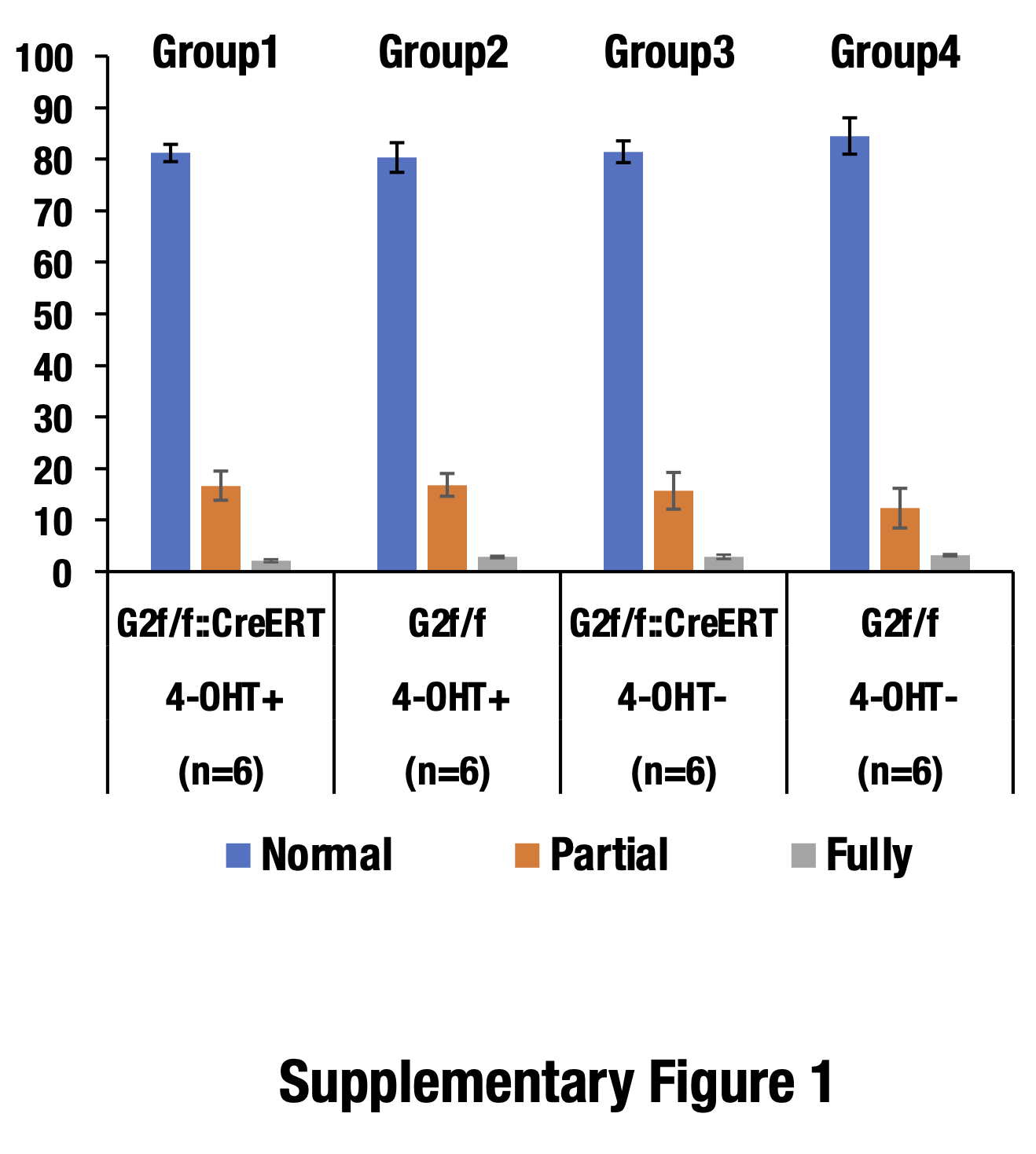

Supplement: S1 Fig — Under normoxic conditions, all group of mice, including Group1 (G2-CKO), exhibited normal vasculature to a similar extent: more than 80% of their pulmonary arterioles were normal, while the remaining arterioles were partially remodeled (12–16%) or fully remodeled in a small number of cases (2–3%). (TIFF) [file pone.0315446.s001.tiff]

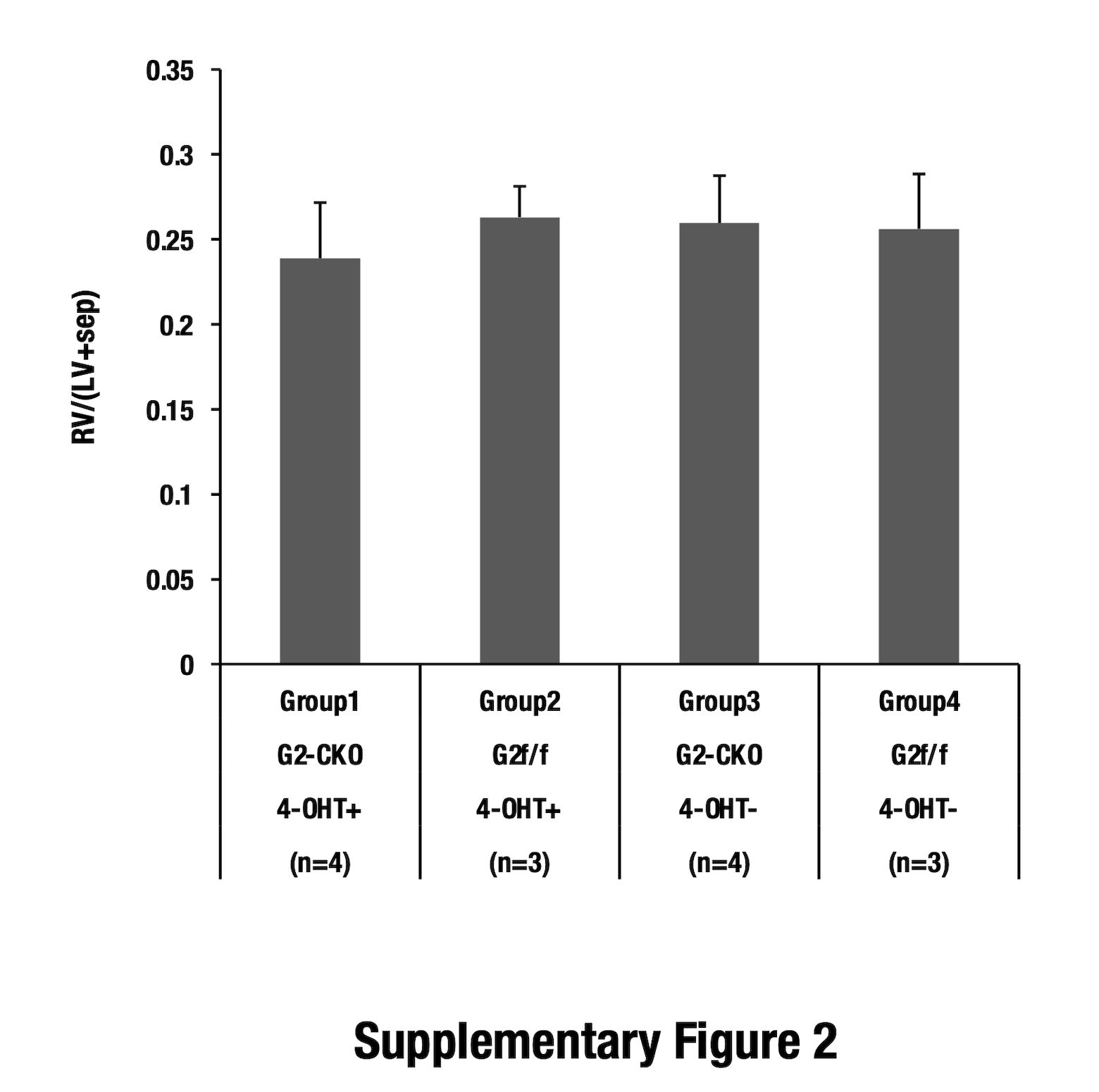

Supplement: S2 Fig — Group1 mice (G2-CKO) show comparable levels of right ventricular wall thickness compared with other group of control mice. (TIFF) [file pone.0315446.s002.tiff]

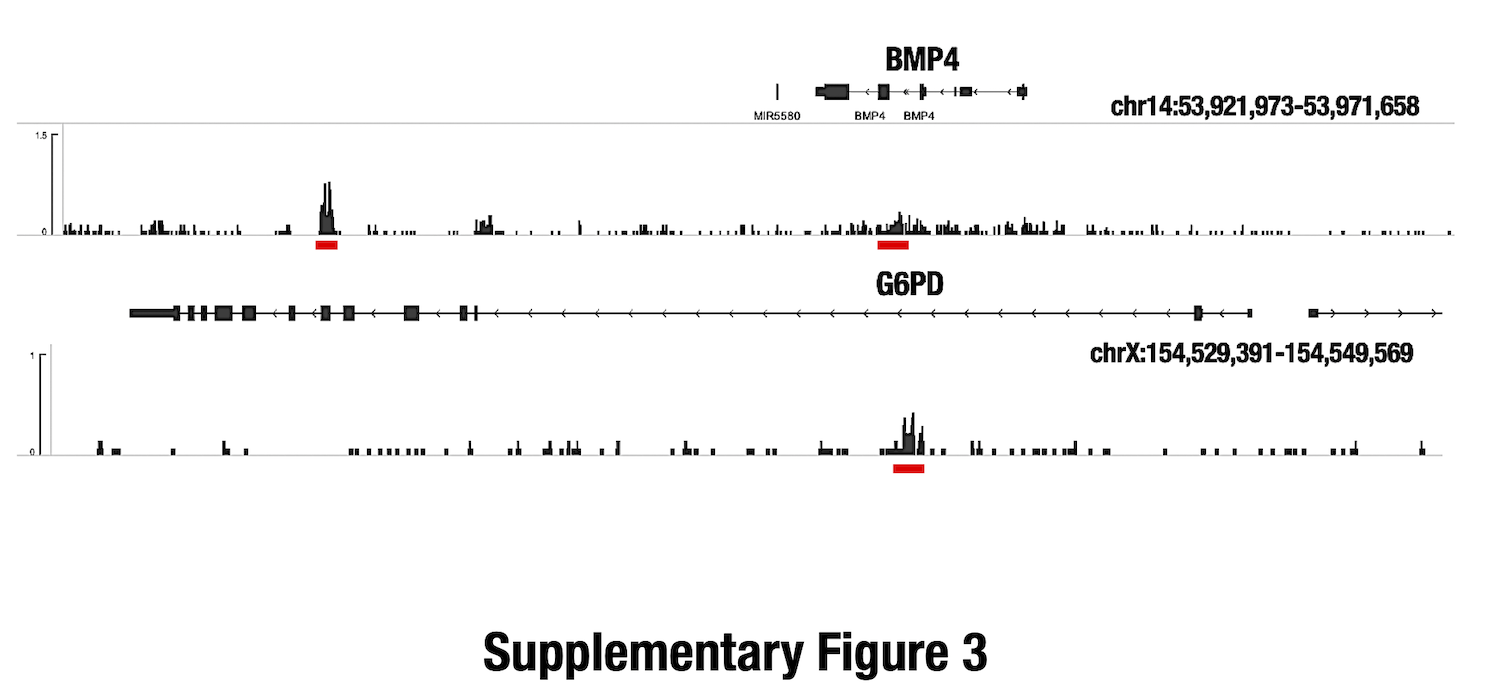

Supplement: S3 Fig — The publicly available ChIP-seq data (GATA2, #SRX070876) are aligned to the human reference genome (hg38) and visualized using the Integrative Genomics Viewer (IGV). (TIFF) [file pone.0315446.s003.tiff]
