# Supplementary material for: GATA2 participates in protection against hypoxia-induced pulmonary vascular remodeling
Source: PLoS One. 2024 Dec 31;19(12):e0315446. doi: 10.1371/journal.pone.0315446 (PMC11687799; doi:10.1371/journal.pone.0315446)
Supplement: Attachment — Submitted filename: Major revision suggesion.docx [file pone.0315446.s004.docx]

In this article, the authors used animal models, tissue immunohistology analyses, cell culture-based analyses, and bioinformatic analyses with public-available data bases to show the important roles that GATA2 may be involved in pulmonary arterial endothelial cells fate and pulmonary vascular remodeling. This research is a pioneer work in further understanding the progression of pulmonary vascular remodeling in pulmonary hypertension. Although the authors have provided some solid evidence to indicate the roles and functions of GATA2, there are major and minor issues that the authors should address.

Major revisions:

1. In Figure 2 and “GATA2 is abundantly expressed in endothelial cells of the pulmonary vasculature” section, the authors used “*Gata2* GFP knock-in mice”. However, the development of the strain is not included in the “Methods” section, and it is recommended to include genotyping results in the supplemental figures. Please also do not forget to include the sequences that are used in the genotyping in Table 1.
2. The authors used the word “control” for many groups (Groups 2, 3 and 4) of mice, but the text did not clearly define which control was used for analysis. It is suggested that the authors just use Group name, instead of using “control”, when presenting the data. For example, what control is used in Line 339
3. In addition to Major revision #2 above, the authors claimed that mice were also kept in normoxic condition; however, these mice were not presented in data. These mice are essential controls to verify the success of hypoxia-induced vascular remodeling. The authors should show that G2f/f mice not bearing the CreERT allele and not treated with 4-OHT developed mild pulmonary vascular remodeling after hypoxia exposure, compared to these mice kept in normoxic condition. This data can be included in supplemental data.
4. In addition to Major revision #2 and #3 above, the authors claimed GATA2 is involved hypoxia-induced pulmonary hypertension; however, what the authors have performed only showed the importance of GATA2 in hypoxia-induced pulmonary vascular remodeling. In order to extend the argument to pulmonary hypertension, right ventricular pressure is required, which will be further supported with right ventricular hypertrophy analysis. The authors should either include the right ventricular pressure data, or correctly conclude their results to be limited to hypoxia-induced pulmonary vascular remodeling, but not pulmonary hypertension.
5. The authors showed that Bmp4 was decreased in G2-CKO lung endothelial cells, but to claim the trend “mirrored” siGATA2 knockdown in HMVEC is not entirely correct. The authors should notice that siGATA2-1 knockdown in HMVEC did very minimum effect on Bmp4 gene expression in HMVEC.

Minor revisions:

1. In Line 186 and Figure 5, there is no statistical analysis.
2. In Figure 7B
   a. The spelling for change is not right
   b. Use Log2(fold change) instead. Explain the meaning of fold change in figure legend.
3. From Line 211 to Line 219, the authors called for Figures 7B and 7C. However, it is not very reader-friendly to call 7C earlier than 7B. Please find a way to make the figure calling in order.
4. Is the induced GATA2 KO in mice global? If it is a global GATA2 KO, did the authors find any phenotype represent myelodysplastic syndromes? Have the authors ever thought of using heterozygous deletion to mimic the GATA2 haploinsufficiency?
5. The authors are suggested to use abbreviations with consistency. For example, Gata2 CKO in Line 349 should be called G2-CKO to avoid confusion.
6. In Line 376, the authors used VWT to represent pulmonary artery wall thickness; however, there is no full name for this abbreviation.
7. From Line 378 to 380, the authors explained to use Ed and Id to calculate wall thickness. In reality, pulmonary artery’s Ed and Id are not consistent. How did the authors average the parameters within one artery is not clear. Please specify.
8. In Line 383 and 384, the authors claimed that they used rabbit anti-GFP and rabbit anti-CD31, while Figure 2C,D showed co-staining of GFP and CD31. If the authors used a specific kit to facilitate this co-staining, please include.
9. From Line 399 to 405, the authors described a TUNEL assay for pulmonary smooth muscle cells. However, there is no data showed in the article.
10. Please also include a brief description of statistical analysis before “Data availability”.
11. Some captions were covered up in Figure 1A
12. Figure 2 legend mentioned panels E and F, but they were not included in the figure.
13. In Figure 3 legend, Line 483, the authors mentioned a “table”, but it is not Table 1. Please include this table after revised.
14. In Figure 4 legend, Line 489, the authors explained they used arrowheads, but there are no arrowheads in Figure 4A
