# Supplementary material for: GATA2 participates in protection against hypoxia-induced pulmonary vascular remodeling
Source: PLoS One. 2024 Dec 31;19(12):e0315446. doi: 10.1371/journal.pone.0315446 (PMC11687799; doi:10.1371/journal.pone.0315446)
Supplement: Attachment — Submitted filename: Response to reviewers(PONE-D-24-36911)1028.docx [file pone.0315446.s005.docx]

PONE-D-24-36911

GATA2 participates in protection against hypoxia-induced pulmonary hypertension

PLOS ONE

**Responses to reviewers
Reviewer #1:** The current research describes the role of the transcription factor GATA2 in in the context of pulmonary hypertension and hypoxia-induced vascular damage. The authors found that GATA2 deficiency predisposes the pulmonary vasculature to damage and fibrosis in response to hypoxia, contributing to pulmonary hypertension. The study topic is of interest but the experiments are poorly designed and lack some key data to support the role of GATA2 in PH model.

**Comment #1-1:** The authors used Rosa26-CreERT to induce systematic GATA2-KO in mice model. It's better to also include cell specific GATA-KO data (such as using Cdh5-CreERT2, Myh11-CreERT2...) to study which cell type contribute to the GATA2 regulation of PH. Moreover, data showing diminished GATA2 expression in the KO mice tissue are needed to demostrate the knockout efficiency.

**Response:** We appreciate the reviewer’s insightful comment. Although we currently do not have vascular cell lineage-specific Cre transgenic lines, we recognize the value of this approach and plan to consider these experiments for future projects.

In the *G2*-CKO mice, the *Gata2* mRNA level in purified lung vascular endothelial cells (CD45−CD31+) was reduced to less than 12% of the level in the control group (Group 3, *G2*f/f::Rosa26-CreERT without 4-OHT) (Fig. 7), highlighting significant knockout efficiency.

**Comment #1-2:** The study don't have the Right ventricular systolic pressure (RVSP) data to support the conclusion of the role of GATA2 in PH. The RVH measuement is flawed since they used formaldehyde-fixed dry hearts instead of fresh tissues. The narrow histological difference shown in Figure 3 may come from gender or age difference of animals since the authors used 10 to 16-week-old Gata2 CKO and control mice in the hypoxia study.

**Response:** We appreciate the reviewer’s critical comments. As noted by both reviewers, the current data do not conclusively demonstrate susceptibility to pulmonary hypertension in *G2*-CKO mice. However, our findings indicate that these mice are predisposed to hypoxia-induced pulmonary vascular remodeling. In response to another reviewer’s suggestion, we have revised the title and conclusion of the manuscript to better reflect these findings.

While measuring the right ventricular systolic pressure (RVSP) would indeed be ideal for assessing pulmonary hypertension, our research facility does not have the equipment necessary for such measurements in small rodents. Consequently, we relied solely on measurement of the right ventricle to left ventricle plus septum weight ratio (RV/LV + S), which has also been utilized in many studies. We have acknowledged this limitation in the Discussion section.

**Comment #1-3:** The whole mice hypoxia study needs normoxia controls to show the effects of hypoxia. The authors mentioned the housing of normoxic mice in the methods section, but no data shown in the manuscript.

**Response;**

Thank you for highlighting this point. We have now organized and analyzed the data on pulmonary vasculature under normoxic conditions. The results indicate that all genotypes of mice display normal vasculature to a similar extent. We have included this new graph as Supplementary Fig. 1.

**Comment #1-4:** In figure 2, the authors show histological data of *Gata2* GFP knock-in mice. However, there is no information about this strain provided in the Methods section. Do this mice strain show different PH phenotypes under hypoxia?

**Response;**

Thank you for this comment. The *Gata2* GFP knock-in (*Gata2*GFP) allele contains the GFP reporter gene inserted at the *Gata2* translational initiation site, disrupting normal GATA2 translation. Homozygous mutants exhibit lethal hematopoietic defects during embryonic development, while heterozygous mice survive to adulthood at expected frequencies. The generation and phenotype of *Gata2*GFP mice were previously described (Suzuki et al., 2006).

We have not yet examined whether the *Gata2*GFP/+ heterozygous mutant mice are susceptible to hypoxia-induced pulmonary vascular remodeling. We have now included the strain information in the Methods section.

**Reviewer #2:** In this article, the authors used animal models, tissue immunohistology analyses, cell culture-based analyses, and bioinformatic analyses with public-available data bases to show the important roles that GATA2 may be involved in pulmonary arterial endothelial cells fate and pulmonary vascular remodeling. This research is a pioneer work in further understanding the progression of pulmonary vascular remodeling in pulmonary hypertension. Although the authors have provided some solid evidence to indicate the roles and functions of GATA2, there are major and minor issues that the authors should address. The authors can also refer to the attached file for revision suggestions.

**Major #2-1:**
1. In Figure 2 and “GATA2 is abundantly expressed in endothelial cells of the pulmonary vasculature” section, the authors used “Gata2 GFP knock-in mice”. However, the development of the strain is not included in the “Methods” section, and it is recommended to include genotyping results in the supplemental figures. Please also do not forget to include the sequences that are used in the genotyping in Table 1.

**Response;**

Thank you for this comment. The *Gata2* GFP knock-in (*Gata2*GFP) allele contains the GFP reporter gene inserted at the *Gata2* translational initiation site, disrupting normal GATA2 translation. Homozygous mutants exhibit lethal hematopoietic defects during embryonic development, while heterozygous mice survive to adulthood at expected frequencies. The generation and phenotype of *Gata2*GFP mice were previously described (Suzuki et al., 2006). The genotyping primer pair for *Gata2*GFP allele is included in Table 1.

**Major #2-2:** The authors used the word “control” for many groups (Groups 2, 3 and 4) of mice, but the text did not clearly define which control was used for analysis. It is suggested that the authors just use Group name, instead of using “control”, when presenting the data. For example, what control is used in Line 339

**Response;**

Thank you for this suggestion. We now refer to the GATA2-deficient mice as *G2*-CKO or Group 1 and specify each control group by its group name (i.e., Groups 2, 3, and 4). We have clarified the genotype and treatment details (i.e., with or without 4-OHT) in the figures. Additionally, we revised the sentence on Line 339 to read: "All mice used in the experiments were matched by weight (15.1–27.0 g) and age (13–15 weeks)."

**Major #2-3:** In addition to Major revision #2 above, the authors claimed that mice were also kept in normoxic condition; however, these mice were not presented in data. These mice are essential controls to verify the success of hypoxia-induced vascular remodeling. The authors should show that G2f/f mice not bearing the CreERT allele and not treated with 4-OHT developed mild pulmonary vascular remodeling after hypoxia exposure, compared to these mice kept in normoxic condition. This data can be included in supplemental data.

**Response;**

Thank you for your valuable comment. In accordance with the reviewer’s suggestion, we have included normoxic controls in Supplementary Fig. 1. The results demonstrate that all mouse genotypes, including *G2*-CKO (Group 1), exhibit nearly normal vasculature under normoxic conditions. Furthermore, these findings highlight that control Groups 2, 3, and 4 still undergo mild pulmonary vascular remodeling following hypoxia exposure.

**Major #2-4:** In addition to Major revision #2 and #3 above, the authors claimed GATA2 is involved hypoxia-induced pulmonary hypertension; however, what the authors have performed only showed the importance of GATA2 in hypoxia-induced pulmonary vascular remodeling. In order to extend the argument to pulmonary hypertension, right ventricular pressure is required, which will be further supported with right ventricular hypertrophy analysis. The authors should either include the right ventricular pressure data, or correctly conclude their results to be limited to hypoxia-induced pulmonary vascular remodeling, but not pulmonary hypertension.

**Response:**

We thank the reviewer for this critical comment. As both reviewers noted, the current data do not conclusively support susceptibility to pulmonary hypertension in the *G2*-CKO mice. However, we demonstrate that the *G2*-CKO mice are prone to hypoxia-induced pulmonary vascular remodeling. In response, we have modified the title and conclusion of the manuscript to better reflect these findings. While measuring the right ventricular systolic pressure (RVSP) would ideally assess the extent of pulmonary hypertension, our research facility lacks the necessary equipment for RVSP measurements in small rodents. Therefore, we relied solely on the measurement of the right ventricle to left ventricle plus septum weight ratio (RV/LV + S), which have also been used in many studies. We have acknowledged this limitation in the Discussion section.

**Major #2-5:** The authors showed that Bmp4 was decreased in G2-CKO lung endothelial cells, but to claim the trend “mirrored” siGATA2 knockdown in HMVEC is not entirely correct. The authors should notice that siGATA2-1 knockdown in HMVEC did very minimum effect on Bmp4 gene expression in HMVEC.
**Response:**

We thank the reviewer for this critical comment. Following this comment, we modified the description in the manuscript.

**Minor #2-1:** In Line 186 and Figure 5, there is no statistical analysis.

**Response:** We added statistical analysis in Fig 5B.

**Minor #2-2:** In Figure 7B
a. The spelling for change is not right
b. Use Log2(fold change) instead. Explain the meaning of fold change in figure legend.
c. From Line 211 to Line 219, the authors called for Figures 7B and 7C. However, it is not very reader-friendly to call 7C earlier than 7B. Please find a way to make the figure calling in order.

**Response:** Thank you for pointing out these oversights. We have corrected the spelling error and updated the figure caption. We have also explained the meaning of fold change in the figure legend: The Log2 ratio of mRNA levels under siGATA2 knockdown compared to those under control random siRNA was calculated to yield the fold change. We also changed the order of the figures so that 7B appears before 7C in the manuscript.

**Minor #2-3:** Is the induced GATA2 KO in mice global? If it is a global GATA2 KO, did the authors find any phenotype represent myelodysplastic syndromes? Have the authors ever thought of using heterozygous deletion to mimic the GATA2 haploinsufficiency?

**Response:** As the reviewer pointed out, the ROSA26-CreERT induces global recombination of the Gata2 flox allele. Our collaborators have demonstrated that the *G2*-CKO mice exhibit fewer red blood cells, white blood cells, and platelets in the peripheral blood. However, the number of splenic macrophages was increased, presumably as a compensatory response (Onodera, 2016). We have recently shown that Gata2 heterozygous mutant mice exhibit some susceptibility to bacterial infections (Takai, 2021), although we have not yet examined whether these mice are susceptible to hypoxia-induced pulmonary vascular remodeling.

**Minor #2-4:** The authors are suggested to use abbreviations with consistency. For example, Gata2 CKO in Line 349 should be called G2-CKO to avoid confusion.

**Response:** Thank you for this valuable comment. We have thoroughly reviewed the manuscript and ensured that all abbreviations, including "G2-CKO," are used consistently to prevent confusion.

**Minor #2-5:** In Line 376, the authors used VWT to represent pulmonary artery wall thickness; however, there is no full name for this abbreviation. From Line 378 to 380, the authors explained to use Ed and Id to calculate wall thickness. In reality, pulmonary artery’s Ed and Id are not consistent. How did the authors average the parameters within one artery is not clear. Please specify.

**Response;** Thank you for pointing out this oversight. We have omitted the description of vascular wall thickness (VWT) because we did not utilize values for the pulmonary artery's wall thickness in this study.

**Minor #2-6:** In Line 383 and 384, the authors claimed that they used rabbit anti-GFP and rabbit anti-CD31, while Figure 2C,D showed co-staining of GFP and CD31. If the authors used a specific kit to facilitate this co-staining, please include.

**Response:** We used chicken anti-GFP (ab13970; Abcam) to conduct GFP-immunohistochemistry. We corrected the related description in the Methods section.

**Minor #2-7:** From Line 399 to 405, the authors described a TUNEL assay for pulmonary smooth muscle cells. However, there is no data showed in the article.

**Response;** We have included the data for the TUNEL assay in Fig 4B and have described it in the relevant section of the results.

**Minor #2-8:** Please also include a brief description of statistical analysis before “Data availability”.

**Response;** Statistical analyses are described in the Methods section and in the legends for each figure.

**Minor #2-9:** Some captions were covered up in Figure 1A

**Response;** We confirmed that all the captions in Figure 1A are fully visible.

**Minor #2-10:** Figure 2 legend mentioned panels E and F, but they were not included in the figure.

**Response;** Thank you for bringing this oversight to our attention. We have now included the additional data in panels in Fig 2E and F.

**Minor #2-11:** In Figure 3 legend, Line 483, the authors mentioned a “table”, but it is not Table 1. Please include this table after revised.

**Response;** The genotype of each group and the number of mice are provided beneath the graph. We have modified the Fig 3 legend accordingly to address the reviewer’s comments.

**Minor #2-12:** In Figure 4 legend, Line 489, the authors explained they used arrowheads, but there are no arrowheads in Figure 4A.

**Response;** We added arrowheads to indicate caspase-positive apoptotic cells in Fig 4A.
